# RiPerC Attenuates Cerebral Ischemia Injury through Regulation of miR-98/PIK3IP1/PI3K/AKT Signaling Pathway

**DOI:** 10.1155/2020/6454281

**Published:** 2020-10-05

**Authors:** Dengwen Zhang, Li Mei, Ruichun Long, Can Cui, Yi Sun, Sheng Wang, Zhengyuan Xia

**Affiliations:** ^1^Department of Anesthesiology, Guangdong Provincial People's Hospital, Guangdong Academy of Medical Sciences, Guangzhou, Guangdong Province, China; ^2^Department of Anesthesiology, Linzhi People's Hospital, Linzhi, Tibet, China; ^3^Department of Anesthesiology, The University of Hong Kong, Hong Kong, SAR, China; ^4^Department of Anesthesiology, Affiliated Hospital of Guangdong Medical University, Zhanjiang, China

## Abstract

**Background:**

Cerebral ischemic stroke is a refractory disease which seriously endangers human health. Remote ischemic perconditioning (RiPerC) by which the sublethal ischemic stimulus is administered during the ischemic event is beneficial after an acute stroke. However, the regulatory mechanism of RiPerC that relieves cerebral ischemic injury is still not completely clear.

**Methods:**

In the present study, we investigated the regulatory mechanism of RiPerC in a rat model of ischemia induced by the middle cerebral artery occlusion (MCAO). Forty-eight adult male Sprague-Dawley (SD) rats were injected intracerebroventricularly with miR-98 agomir, miR-98 antagomir, or their negative controls (agomir-NC, antagomir-NC) 2 h before MCAO or MCAO+RiPerC followed by animal behavior tests and infraction volume measurement at 24 h after MCAO. The expression of miR-98, PIK3IP1, and tight junction proteins in rat hippocampus and cerebral cortex tissues was detected by quantitative polymerase chain reaction (qPCR) and Western blot (WB). Enzyme-linked immunosorbent assay (ELISA) was used to assess the IL-1*β*, IL-6, and TNF-*α* levels in the rat serum.

**Results:**

The results showed that in MCAO group, the expression of PIK3IP1 was upregulated, but decreased after RiPerC treatment. Then, we found that PIK3IP1 was a potential target of miR-98. Treatment with miR-98 agomir decreased the infraction volume, reduced brain edema, and improved neurological functions compared to control rats. But treating with miR-98 antagomir in RiPerC group, the protective effect on cerebral ischemia injury was canceled.

**Conclusion:**

Our finding indicated that RiPerC inhibited the MCAO-induced expression of PIK3IP1 through upregulated miR-98, thereby reducing the apoptosis induced by PIK3IP1 through the PI3K/AKT signaling pathway, thus reducing the cerebral ischemia-reperfusion injury.

## 1. Introduction

Cerebral ischemic stroke is a refractory disease which seriously endangers human health. It is characterized by high incidence, high disability, and high mortality. Cerebral ischemic injury is one of the serious perioperative complications, which can cause neurological dysfunction and is an important cause of death and disability of patients. Perioperative general anesthesia, postoperative dehydration, bed rest, and other factors can all increase the risk of cerebral ischemia. Statistical studies show that the risk of stroke in general surgical patients is up to 0.1-3%, and the risk of stroke in patients with complex heart surgery is up to 10% [1, 2]. Ischemic stroke is caused by insufficient blood supply to the brain and is characterized by hypoxia, excitotoxicity, and inflammation, which ultimately lead to neuronal cell death [3, 4]. Since neurons are difficult to regenerate, the research on how to reduce the death and apoptosis of neurons and increase the tolerance of neurons to ischemia is the main direction of the current research on the treatment of cerebral ischemic injury.

Recent studies have found that during cerebral ischemia, transient ischemic reperfusion stimulation in the distal limb can also reduce cerebral ischemia reperfusion injury. This protective effect is known as remote ischemic perconditioning (RiPerC) [5, 6]. This is different from previously reported ischemic preconditioning (IPC) and drug preconditioning, including opioid agonists, inhaled anesthetics, and adenosine that can reduce ischemic reperfusion injury [7, 8]. Those preconditioning measures must be implemented before the occurrence of ischemia. However, the occurrence of clinical ischemia is often unpredictable, which restricts the clinical application of these preconditioning methods. The very important advantage of RiPerC is that it can be carried out during the occurrence of ischemia without the support of very complex technical conditions, so it should have a broader clinical application prospect [9, 10]. Therefore, the mechanism by which RiPerC operation alleviates cerebral ischemia injury deserves further investigation.

The phosphatidylinositol 3-kinase/protein kinase B (PI3K/Akt) signaling pathway, involved in the regulation of cell proliferation and differentiation, has been documented to protect neural stem cells (NSCs) against oxidative damage [11, 12]. Activation of the PI3K/Akt signaling pathway has been implicated in neuroprotective effects of various agents against ischemia reperfusion injury [13, 14]. In the light of the findings cited above, we hypothesized that the PI3K/Akt signaling pathway may account for the neuroprotective effects of RiPerC operation.

## 2. Materials and Methods

### 2.1. Animals

Forty-eight adult male Sprague-Dawley (SD) rats weighing 250-300 g were randomly divided into 7 groups: sham group (sham operated rats without other treatment; *n* = 12), MCAO group (modeled rats; *n* = 6), RiPerC group (modeled rats with RiPerC operation; *n* = 6), MCAO-NC agomir group (modeled rats with lateral cerebroventricular injection of NC agomir; *n* = 6), MCAO-miR-98 agomir group (modeled rats with lateral cerebroventricular injection of miR-98 agomir; *n* = 6), RiPerC-NC antagomir group (modeled rats with lateral cerebroventricular injection of NC antagomir; *n* = 6), and RiPerC-miR-98 antagomir group (modeled rats with lateral cerebroventricular injection of miR-98 antagomir; *n* = 6). The rats were housed in a clean animal room with room temperature at 22 ± 2°C, the relative humidity at 60%, and 12 h day/night cycle. The litter was changed every day to avoid infection. All procedures involving animals were performed according to the USA Care and Use of Laboratory Animals. Animals were purchased from the Experimental Animal Center of Sun Yat-sen University (Guangzhou, China). This study was approved by the Animal Ethics Committee of Guangzhou Forevergen Medical Experimental Animal Center.

### 2.2. Establishment of Rat MCAO Model

Middle cerebral artery occlusion (MCAO) rat model was established according to the reference [15, 16]. Rats were deeply anesthetized with an intraperitoneal injection of chloralic hydras (0.4 g/kg, J1516063, Aladdin Biochemical Technology Co., Ltd. Shanghai, China). A blunt dissection was performed under a stereomicroscope (Stemi 2000, Carl Zeiss) to expose the left common carotid artery (CCA), then gradually expose CCA bifurcation, external carotid artery (ECA), and internal carotid artery (ICA), until near skull base, followed by ligation of the ipsilateral CCA proximal end and external carotid artery and clamping of the CCA and ICA with arterial clamp. This was followed by a small incision in the ECA between permanent and temporary sutures, in which a 5-0 surgical nylon filament with a round silica gel tip (0.34 ± 0.02 mm in diameter) (L3400, Guangzhou Jialing Biotechnology Co., Ltd., Guangzhou Jialing Biotechnology Co. LTD, Guangzhou, China) was inserted into the ICA approximately 18-20 mm beyond the carotid bifurcation, thereby occluding the origin of the middle cerebral artery. Loosen the arteriole clip and suture the skin. The end of the occlusion line will be slightly exposed to the skin 1 cm. After 2 hours of MCAO, the rat was allowed to recover for 1 day. The sham operation group was performed with the same surgical procedure except for the ligation and the strand placement. Rectal temperature was maintained at 37.0°C during and after surgery with a temperature control heating pad.

RiPerC operation was carried out during MCAO ischemia. In RiPerC group, a tourniquet was applied around the right hind-limb just below the level of the inguinal ligament for 3 × 10 minutes with 10-minute intermittent reperfusion periods. This process was performed with MCAO at the same time.

### 2.3. Intracerebroventricular Injection

MiR-98 agomir (agomir-98, RiboBio, Guangzhou, China), which is a chemically modified double-stranded miRNA-98 that mimics the endogenous miR-98 was injected into hippocampus by intracerebroventricular (ICV) injection to establish the overexpression of miR-98 in the rat, whilst miR-98 antagomir (antagomir-98, RiboBio) is a chemically modified single-stranded miRNA and perfectly complementary to the miR-98 sequence. Through binding to the miR-98, miR-98 antagomir can inhibit the function of miR-98. To knockdown miR-98 expression in rat, miR-98 antagomir was injected into hippocampus by ICV injection according to the manufacturer's instructions. Briefly, agomir-98, antagomir-98, agomir-NC, and antagomir-NC (0.8 nmol dissolved in 4 *μ*L PBS; RiboBio) were applied 3 days before MCAO. The injections were performed as previously described [17]. Rats were anesthetized and positioned lying prone in a stereotactic head frame (RWD Life Science, China). A scalp incision was made along the midline, and a burr hole was drilled into the right side of the skull (0.5 mm posterior and 1.0 mm lateral to the bregma). AgomiR-98, antagomiR-98, agomir-NC, and antagomir-NC were microinfused into right lateral ventricles through a Hamilton syringe (2.5 mm vertically), which was driven by a microinfusion pump (KDS 310, KD Scientific) with 0.2 *μ*L/min. The needle was left in place for an additional 5 min after injection to prevent possible leakage and was slowly withdrawn within 4 min. After the needle was removed, the burr hole was sealed with bone wax, the incision was closed with sutures, and the rats were allowed to recover.

### 2.4. Rat Neurological Function Score

According to Longa's 5-point scoring method [18], scoring was started from the time when the MCAO rat first recovered completely. 0 point, normal without neurological deficit; 1 point, unilateral forelimb cannot be straightened after rising; 2 points, the body tilted to one side when the rat was crawling forward; 3 points, the rat's crawling body fell to the side; 4 points, coma or cannot crawl spontaneously.

### 2.5. 2,3,5-Triphenyltetrazolium Hydrochloride (TTC) Staining

After 24 h reperfusion, animals were sacrificed by common carotid perfusion fixation with cold Tris-buffered saline under isoflurane anesthesia. The brain was immediately removed and sectioned into five coronal slices (2 mm in thickness) using a brain-cutting matrix. The brain slices were stained with 1% 2,3,5-triphenyltetrazolium chloride (TTC; Sigma-Aldrich Pty Ltd, Australia) at 37°C in the dark for 30 min. Noninfarcted tissues were stained (red), and infarct tissues were not stained (white). Then, brain sections were fixed in 2% paraformaldehyde and photographed with a digital camera (Canon IXUS175, Tokyo, Japan). The percentage of infarct volume was analysed using Image J software 1.50i by calculating the infarct volume ratio. Briefly, the infarct volume was calculated as a percentage of the entire brain adjusted for edema using modified Swanson calculation.

### 2.6. Quantitative Polymerase Chain Reaction (PCR)

Total RNA was extracted by TRIzol (Invitrogen, Carlsbad, CA, USA) using an RNA extraction kit. Complementary deoxyribose nucleic acid (cDNA) was synthesized by reverse transcription. The levels of mature miR-98 were determined using a stem-loop real-time PCR system with TaqMan Universal Master Mix II (Ambion, CA, USA). The miR-98 levels were normalized to those of U6 snRNA. The level of PIK3IP1 expression was detected by quantitative PCR. The sequences of the primers are listed below: miRNA-98 (Forward) 5′-TGAGGTAGTAAGTTGTATTGTT-3′; U6 (Forward) 5′-GCAAATTCGTGAAGCGTTCC-3′; PIK3IP1 (Forward) 5′-AGAGACCACTTCCGGTGACA-3′; (Reverse) 5′-ACACGTAGCCCAAAGTTCCC-3′; *β*-actin (Forward) 5′-AGATCAAGATCATTGCTCCTCCT-3′; (Reverse) 5′-ACGCAGCTCAGTAACAGTCC-3′. The fold change in relative miRNA expression was determined using the 2^−ΔΔCt^ method, as described previously [19].

### 2.7. Enzyme-Linked Immunosorbent Assay (ELISA)

Enzyme-linked immunosorbent assay (ELISA) was used to assess the IL-1*β*, IL-6, and TNF-*α* levels in the rat serum. IL-1*β*, IL-6, and TNF-*α* were measured with commercial ELISA kits (Boster Biosciences Co., Wuhan, China) according to the manufacturer's instructions. A microplate reader (Infinite M200 PRO, Tecan, Switzerland) was used to assess the OD value.

### 2.8. Luciferase Reporter Assay

The wild type of the 3′ untranslated region (3′UTR) of the PIK3IP1 gene (including miR-98 binding sites) and mutant 3′UTR of the PIK3IP1 gene were synthesized by Guangzhou HYY Biotechnology Co. LTD (Guangzhou, People's Republic of China) and cloned into the downstream portion of the psiCHECK-2 vector (Promega) to generate PIK3IP1–WT and mutant PIK3IP1 (PIK3IP1–MUT), which were confirmed by sequencing. For the luciferase reporter assay, 293T cells were seeded in 24-well plates at a density of 2 × 10^4^ cells per well. When the cells reached 70% confluency, they were cotransfected with either PIK3IP1–WT (100 ng) or PIK3IP1–MUT (100 ng) and miR-98 mimics (100 nM) or miR-NC mimics (100 nM). Forty-eight hours later, cells were harvested and assayed using the Dual-Luciferase Reporter Assay System (LF005, FulenGen, Guangzhou, China) according to the manufacturer's instruction. Each experiment was independently repeated 3 times.

### 2.9. Western Blot Assay

Rats were sacrificed, and the cerebral cortex and hippocampus were collected. Whole-cell protein was prepared from the ischemic cortices divided from left cerebral hemisphere. In brief, brain tissues were homogenized in RIPA buffer (P1003B, Beyotime, Wuhan, China) containing PMSF (ST506, Beyotime, Wuhan, China) and then sonicated on ice. After centrifugation, the supernatant was collected for Western blot assay. An aliquot of 10 *μ*g/mg protein from each sample was separated by SDS-PAGE and then transferred onto a nitrocellulose membrane. The membrane was blocked with 5% nonfat milk in TBST for 2 h (pH 7.4) and then incubated with primary antibodies against PI3KIP1 (1 : 1000; sc-365777, Santa Cruz), *β*-catenin (1 : 1000; BM0627, BOSTER), Bcl-2 (1 : 1000; ab32124, Abcam), Bax (1 : 1000; ab32503, Abcam), caspase 9 (1 : 1000, ab32539, Abcam), AKT (1 : 1000, 71632S, Cell Signaling Technology), p-AKT (1 : 1000, 9271S, Cell Signaling Technology), PI3K (1 : 1000, 4249S, Cell Signaling Technology), and p-PI3K (1 : 1000, 17366S, Cell Signaling Technology) at 4°C overnight. After incubation with secondary antibody for 2 h at room temperature, visualization was done with a chemiluminescence imaging analysis system (5200, Tanon, China). The gray value of PI3KIP1, *β*-catenin, Bcl-2, Bax, Caspase 9, AKT, p-AKT, PI3K, and p-PI3K was measured with Image-J software and normalized to that of *β*-actin as the relative protein expression.

### 2.10. Terminal Deoxynu-Cleotidyl Trans/Erase- (TDT-) Mediated dUTP-Biotin Nick End-Labeling (TUNEL) Staining

At 24 h after the operation, the brain tissue was fixed with 4% paraformaldehyde, routinely dehydrated, paraffin-embedded, and then sliced at a thickness of 3 *μ*m. Afterward, the section was dewaxed and hydrated and boiled. The section was then treated with 0.01 mol/L citrate buffer (pH 6.0), and the DNA fragment labeling was performed. The staining was conducted according to the instructions of the TUNEL Kit (Bollingman, Beijing, China). The results of the experiment were shown as the number of cells with apoptotic nuclei and the cells' total number in each high-power field of view (six high-power fields per section). The apoptotic index (AI) is the apoptotic nuclei's number out of 100 nuclei. The average value of AI was calculated as TUNEL positive cells/total cell number × 100%. Each experiment runs in triplicate.

### 2.11. Statistical Analyses

The analysis of the data was performed using the GraphPad Prism software. Multiple comparisons were statistically analyzed with one-way analysis of variance (ANOVA) followed by the Tukey method or nonparametric test. The data are presented as means ± SEM, and a *P* value < 0.05 represents statistical significance.

## 3. Result

### 3.1. RiPerC Protects Rat against Ischemic Brain Injury at the Structural and Functional Levels

**Figure 1:**
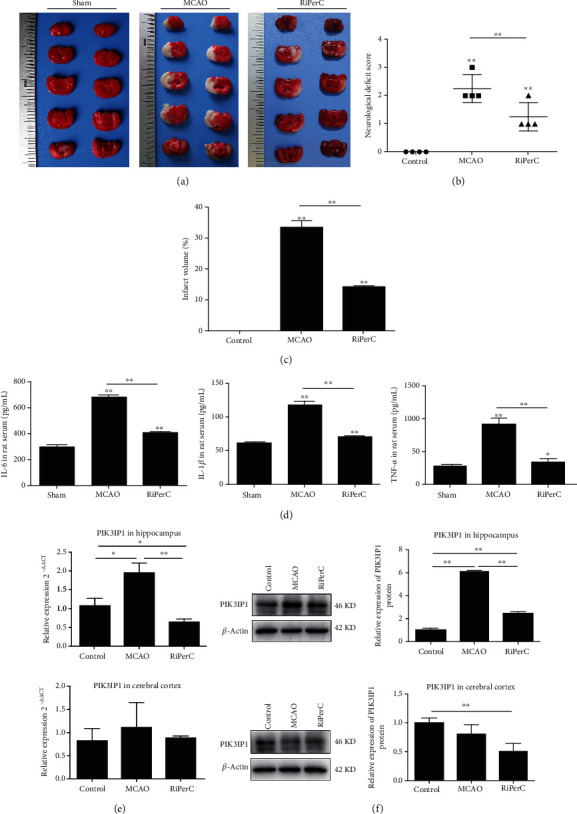
RiPerC reduced acute infarct damage in rat following MCAO. (a) Representative images of TTC staining in brain sections collected from rat receiving RiPerC operation at 1 day after reperfusion. (b) The neurological deficit score was assessed by the Longa scale scoring system at 1 day after reperfusion. (c) Quantitative data regarding the effects of the RiPerC operation on cerebral infarction as assessed by TTC histology at 1 day (*n* = 4 per group). (d) The expression of IL-1*β*, IL-6, and TNF-*α* in rat serum by ELISA. (e) The expression of PI3KIP1 in hippocampus and cerebral cortex by qPCR and WB.

We investigated the effects of RiPerC on ischemic brain injury. The rat received a RiPerC operation during MCAO operation. Compared to the MCAO group alone, the combination of RiPerC and MCAO operation displayed lower neurological deficit score (Figure 1(b)) and infarct volume (Figures 1(b) and 1(c)).

Immunity and inflammation are key elements in the pathophysiology of stroke; we detected the inflammation factors by ELISA. Compared with the sham group, the expression of IL-1*β*, IL-6, and TNFa was all upregulated in MCAO group, while the expression of IL-1*β*, IL-6, and TNFa was downregulated with RiPerC treatment (Figure 1(d)).

Some studies have pointed out that the PI3K/Akt signalling pathway participates in neuronal injury [20, 21], which has been reported to suppress neuronal apoptosis [22]. Given the fact that PIK3IP1 downregulates PI3K activity, we detected the mRNA and protein levels of PIK3IP1. In MCAO group, the mRNA and protein expression of PIK3IP1 was upregulated in both hippocampus and cerebral cortex. However, the mRNA and protein expression of PIK3IP1 was decreased with RiPerC treatment (Figures 1(e) and 1(f)).

### 3.2. PIK3IP1 Is a Potential Target of miR-98

**Figure 2:**
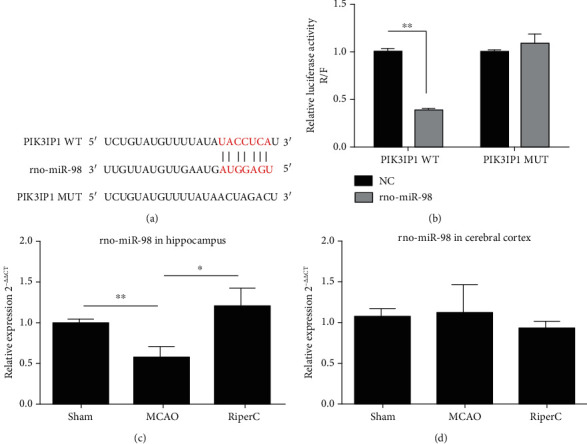
PIK3IP1 3′-UTR was directly targeted by miR-98. (a) Schema of the WT and mutated PIK3IP1 3′-UTR indicating the interaction sites between miR-98 and the 3′-UTR of PIK3IP1. (b) Dual luciferase assay in HEK293T cells cotransfected with the miR-98 mimic and reporter vectors containing either the wild-type or mutated 3′-UTR of PIK3IP1. (c) The miR-98 in rats hippocampus tissue detected by qPCR. (d) The miR-98 in rats cerebral cortex tissue detected by qPCR.

To explore the signaling pathway of PIK3IP1-modulated neuroinflammation, we identified the potential miRNA which targets of PIK3IP1 by using the computer algorithm (http://www.microrna.org/microrna/home.do). Among all of the predicted miRNA, miR-98 was chosen as a candidate because it is reported to be involved in MCAO. In addition, the seed sequence of miR-98 displays perfectly complementary matching with the 3′UTR of the PIK3IP1 gene (Figure 2(a)). To obtain direct evidence that PIK3IP1 was a target of miR-98, the fragment of the PIK3IP1 3′-UTR containing the nucleotides complementary to miR-98 was cloned into a luciferase reporter plasmid (psiCHECK-2), such that the PIK3IP1 3′-UTR was placed downstream of the luciferase reporter gene. Plasmids containing either the wild-type or mutant PIK3IP1 3′-UTR were then cotransfected with the miR-98 mimic in HEK 293T cells. The miR-98 mimic inhibited luciferase activity in the HEK 293T cells transfected with the wild-type PIK3IP1 3′-UTR, but such reduction of luciferase activity was cancelled when the miR-98 binding site was mutated, indicating that PIK3IP1 is a direct target gene of miR-98 (Figure 2(b)).

Considering that miR-98 plays an important regulatory role in cerebral ischemic injury, we used qRT-PCR to assess the expression levels of miR-98 in rat hippocampus tissue and cerebral cortex tissue of rats in MCAO group and RiPerC group. Compared to the sham-operated rat, rat receiving MCAO displayed a significant decrease in level of miR-98 in the hippocampus tissue at 24 h after reperfusion. Furthermore, relative to the MCAO-operated brain, miR-98 level was remarkably enhanced in the hippocampus tissue after RiPerC operation (Figure 2(c)). But the expression of miR-98 was not significantly different in cerebral cortex tissues (Figure 2(d)).

### 3.3. miRNA-98 Expression in Hippocampus Tissue Play a Key Role in Cerebral Ischemic Injury

**Figure 3:**
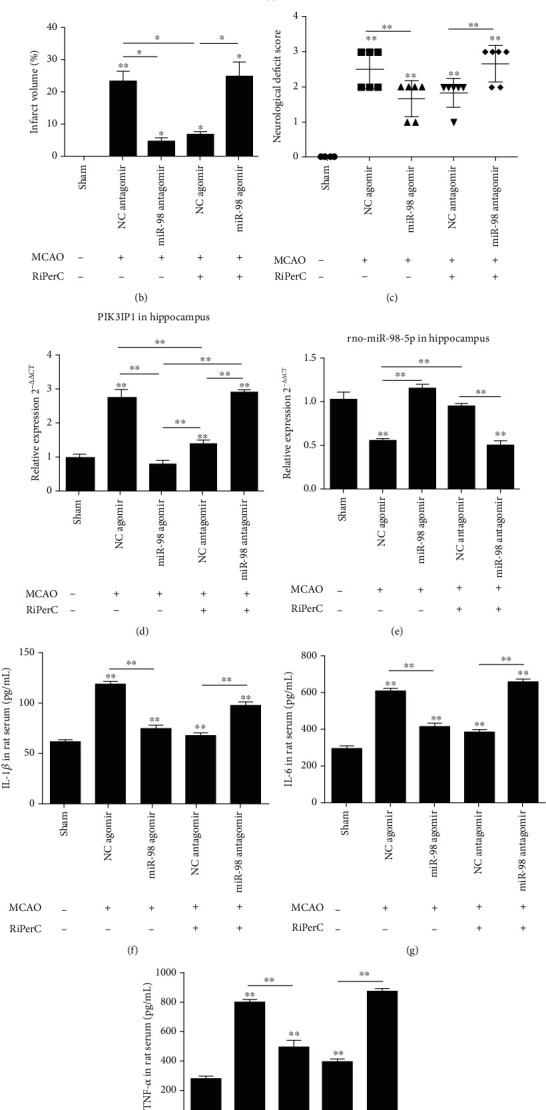
The miRNA-98 expression in hippocampus tissue plays a key role in cerebral ischemic injury. (a) Representative images of TTC staining in brain sections collected from rat receiving RiPerC operation or ICV injection at 1 day after reperfusion. (b) The neurological deficit score was assessed by the Longa scale scoring system at 1 day after reperfusion. (c) Quantitative data regarding the effects of the RiPerC operation on cerebral infarction as assessed by TTC histology at 1 day (*n* = 4 per group). (d, e) The expression of PI3KIP1 and miR-98 in hippocampus detected by qPCR. (f–h) The expression of IL-1*β*, IL-6, and TNF-*α* in rat serum by ELISA.

To investigate the overexpression of miR-98 is the role of MCAO on cerebral ischemic injury. The rat received an ICV infusion of either the miR-98 agomir or control NC agomir 3 days prior to MCAO. Compared to the sham group, MCAO suppressed the level of miR-98, while the miR-98 agomir injection enhanced the level of miR-98 to similar extent with the sham group. The result showed that miR-98 agomir displayed significantly smaller infarct volumes (Figures 3(a) and 3(b)), lower neurological deficit (Figure 3(c)), and lower levels of inflammatory cytokines in serum when compared with NC-agomir group (Figures 3(f)–3(h)). The qPCR measurement confirmed that the miR-98 agomir injection increased postischemic induction of miR-98 expression in hippocampus, 24 h after reperfusion in MCAO rat. Compared to the NC-agomir, the expression of miR-98 was significantly upregulated 2.054 times (*P* < 0.05, Figure 3(e)), and the expression of PIK3IP1 was significantly downregulated 0.718 times (*P* < 0.05, Figure 3(d)). The protein expression of PIK3IP1 detected by WB showed the same results.

To investigate whether knockdown of miR-98 cancels the protective effect of RiPerC on cerebral ischemic injury, the rat received an ICV infusion of miR-98 antagomir and NC antagomir 3 days prior to MCAO and RiPerC operation. The results showed that miR-98 antagomir suppressed the RiPerC-mediated induction of miR-98. miR-98 antagomir canceled the protective effect of RiPerC, as evidenced by increased infarct volumes (Figures 3(a) and 3(b)), enhanced neurological deficit (Figure 3(c)), and greater levels of inflammatory cytokines (Figures 3(f)–3(h)), suggesting miR-98 mediates the effect of RiPerC on cerebral ischemic injury. The result of qPCR showed that the miR-98 expression in the RiPerC+miR-98 antagomir group was lower than that in the RiPerC+NC antagomir group (*P* < 0.05, Figure 3(e)), and the PIK3IP1 expression in the RiPerC+miR-98 antagomir group was higher than that in the RiPerC+NC antagomir group (*P* < 0.05, Figure 3(d)).

### 3.4. miR-98 Mediates the Antiapoptotic Effect of RiPerC in MCAO Model

**Figure 4:**
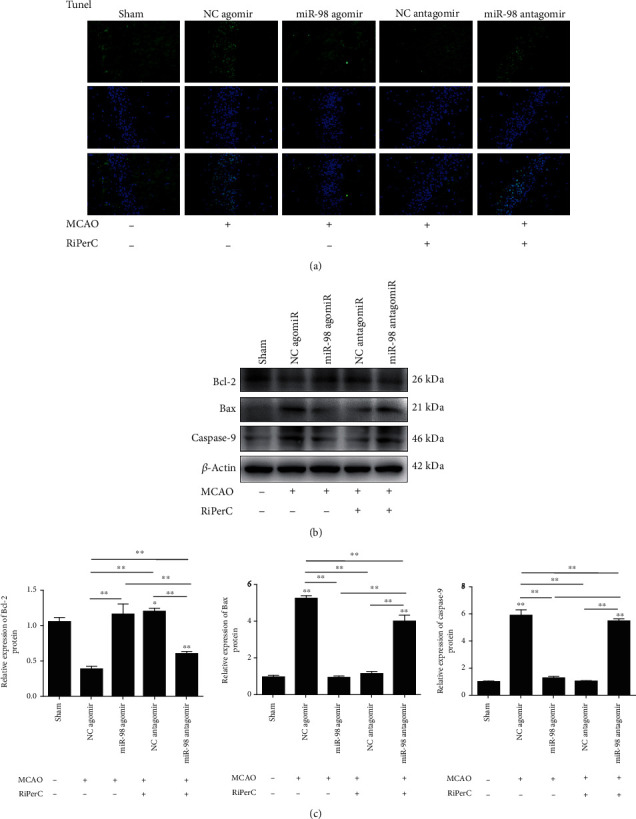
Suppression of miR-98 by decreased the neuronal apoptosis in RiPerC. (a) Brain cell apoptosis was detected by TUNEL assay. (b, c) The Western blot assay to measure the expression of apoptosis-related proteins in rat hippocampus tissue. ^∗^*P* < 0.05, ^∗∗^*P* < 0.01.

TUNEL staining was then performed to determine apoptosis of hippocampus in each rat group, the results indicated that the apoptosis rate in the MCAO injected with miR-98 agomir was lower than the MCAO injected with NC agomir group (Figure 4(a)). Relative to the RiPerC injected with NC antagomir group, the apoptotic cells elevated in the RiPerC injected with miR-98 antagomir group (Figure 4(a)). Furthermore, Western blot assay was employed to detect the apoptosis-related proteins caspase-9, Bax, and Bcl-2 expression. The findings (Figure 4(b)) indicated that significant upregulation was detected in caspase-9 and Bax, and Bcl-2 was downregulation between the MCAO and the sham groups (all *P* < 0.05). In comparison to the MCAO injected with NC agomir group, caspase-9 and Bax reduced in the MCAO injected with miR-98 agomir group, accompanied by reduced Bcl-2 (all *P* < 0.05).

Compared with the RiPerC+NC antagomir group, TUNEL staining showed that the apoptosis rate in the RiPerC injected with miR-98 antagomir was significantly upregulated (Figure 4(a)). The protein expression of caspase 9, Bax, and Bcl-2 was detected by WB; the result showed that caspase 9 and Bax in RiPerC+miR-98 antagomir were upregulated, and Bcl-2 in RiPerC+miR-98 antagomir was downregulated when compare with RiPerC+NC antagomir.

### 3.5. PI3K/Akt Signaling Pathway Was Modulated by miR-98/PIK3IP1 Axis in Cerebral Ischemic Injury In Vivo

**Figure 5:**
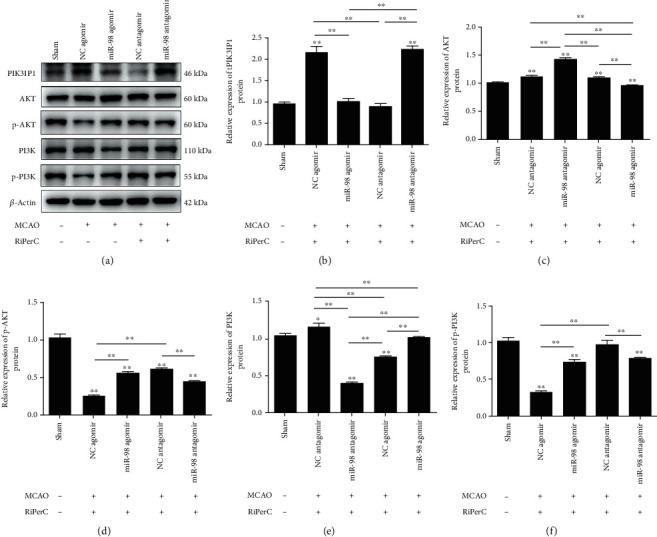
PI3K/Akt signaling pathway was modulated by miR-98/PIK3IP1 axis in cerebral ischemic injury in vivo. (a–f) The Western blot assay to measure the expression of PIK3IP1, AKT, p-AKT, PI3K, and p-PI3K proteins in rat hippocampus tissue. ^∗^*P* < 0.05, ^∗∗^*P* < 0.01.

To further explore the molecular mechanism of miR-98 in regulating the development of cerebral ischemic injury, we investigated whether PI3K/Akt signaling pathway was involved in the progression of cerebral ischemic injury regulated by miR-98/PIK3IP1 axis. Western blot assay was carried out to detect the downstream genes of PI3K/Akt pathway in the cerebral ischemia injury, including PIK3IP1, PI3K, p-PI3K, Akt, and p-Akt (Figure 5(a)). The findings displayed that the phosphorylation of PI3K and Akt was decreased markedly after MCAO operation. Treatment with miR-98 agomir and RiPerC operation significantly increased the expression of p-PI3K and p-Akt. However, RiPerC operation group treatment with miR-98 antagomir decreased the expression of p-PI3K and p-Akt (Figures 5(b)–5(f)). These results revealed that miR-98 promoted, whereas PIK3IP1 inhibited the activation of PI3K/Akt pathway in rat hippocampus after cerebral ischemic injury, suggesting that miR-98 could activate the PI3K/Akt pathway by suppressing PIK3IP1 in cerebral ischemic injury.

## 4. Discussion

RiPerC by which the sublethal ischemic stimulus is administered during the ischemic event is beneficial after an acute stroke [23]. Both remote ischemic pre- and perconditioning have now been proven effective in animal models, and remote perconditioning was even found to be superior to preconditioning [24, 25]. A potent endogenous protective mechanism of RiPerC is that ischemia induced in one organ leads to ischemic tolerance in other organs [26]. However, the regulatory mechanism of RiPerC relieve cerebral ischemic injury is still not completely clear.

PIK3IP1 is a transmembrane protein that possesses an intracellular domain homologous to the p85 regulatory subunit of PI3K, which downregulates PI3K activity by binding to a PI3K subunit through a specific domain [27]. It is abundantly expressed in many tissues, including the heart, liver, brain, and lung. It is reported that the overexpression of PIK3IP1 in mouse hepatocytes leads to a reduction in PI3K signaling and the suppression of hepatocyte carcinoma development [28]. PIK3IP1 participates in the PI3K pathway, which is in many cellular functions such as T cell activation, carcinogenesis, and apoptosis [29, 30]. Several studies have shown that silencing of PIK3IP1 increases PI3K activity in basal conditions [27]. In the present study, RiPerC significantly reduced neurobehavioral deficits and reduced the percentage of infarction volume, which is associated with downregulation of PI3KIP1 in ischemic perconditioning. The research of Shaurya et al. has been reported that let-7 represses PIK3IP1 in hypoxia myocytes in vitro [31]. Studies also showed that the expression patterns of a series of microRNAs in ischemic tissues had changed dramatically [32, 33]. To explore the signaling pathway of PIK3IP1-modulated neuroinflammation, we identified the potential miRNA which targets PIK3IP1 by using the microRNA program (http://www.microrna.org/microrna/home.do). Among the predicted miRNAs, miR-98 was chosen as a candidate because it is reported to be involved in MCAO [34]. There have been several other studies reporting the altered expression of miR-98 during the ischemia injury [35, 36]. A study has shown that let-7/miR-98 regulated Fas expression and the sensitivity of Fas-mediated apoptosis [37]. In this study, we also found that miR-98 was downregulated in MCAO model group, then upregulated when rat received RiPerC operation. Meanwhile, our result indicated that RiPerC operation and the overexpression of miR-98 can reduce the apoptosis rate induced by cerebral ischemic injury in hippocampus tissue.

In order to explore whether miR-98 acts protective effects on cerebral ischemic injury, we made use of miR-98 agomir/antagomir by ICV infusion to elevate miR-98 expression in rat hippocampus prior to MCAO/RiPerC+MCAO operation. As expected, overexpression of miR-98 significantly reduced the cerebral ischemic injury compared with the MCAO group, and inhibition of miR-98 significantly offsets the cerebral ischemic injury relief by RiPerC treatment. In addition, overexpression of miR-98 administration significantly decreased the number of TUNEL-positive cells in hippocompus tissue. These results indicated the protective effect of miR-98 in cerebral ischemic injury. Furthermore, the results showed that RiPerC relieve cerebral ischemic injury might be regulated through miR-98.

Studies have indicated that inflammatory response runs through the pathological development of cerebral ischemia-reperfusion and is one of the main causes of neuronal death [38]. Elevated proinflammatory cytokines aggregate a large number of inflammatory cells which secrete excessive inflammatory cytokines and aggravate brain damage [39]. Mo et al. found that OGD/R induced the expression of IL-1*β*, IL-6, and TNF-*α* in the supernatant of microglia [40]. In the results of Zhang et al., inflammatory factors, such as TNF-*α*, IL-1*β*, IL-6, iNOS, CD32, and CD68, were markedly upregulated in the ischemic striatum at 24 h after reperfusion in mice following MCAO [41]. In our study, we detected the proinflammatory cytokines IL-1*β*, IL-6, and TNF-*α* by ELISA. The results showed that IL-1*β*, IL-6, and TNF-*α* levels were increased in MCAO group, which is in line with the reports of Mo et al. and Zhang et al. Moreover, the levels of IL-1*β*, IL-6, and TNF-*α* were decreased when rats received RiPerC operation and miR-98 agomir injection.

The PI3K/AKT signaling pathway can inhibit apoptosis through the following pathways [42]: (1) increase the activity of antiapoptotic protein Bcl-2; (2) inhibition of the activation of aspartic acid-specific cysteine protease family members and inhibition of apoptosis induced by caspase-9; (3) inhibition of the expression of proapoptotic genes; (4) ATK activates IKK*α*, leading to the degradation of NF-*κ*B inhibitor I*κ*B, which releases NF-*κ*B from the cytoplasm for nuclear translocation, activating its target genes and promoting cell survival. In our study, we find that miR-98 agomir and RiPerC operation significantly increased the expression of Bcl-2 and decreased the expression of Bax and caspase-9. When suppressing the expression of miR-98 in RiPerC group, the regulations of Bcl-2, Bax, and caspase-9 were all reversed. The results of apoptotic cell detected by TUNEL stain in each group were consistent with Bax and caspase-9 expression.

Studies have shown that PIK3IP1 can bind to PI3K, downregulate the activity of AKT, and inhibit the activation of AKT [28]. Our findings displayed that the phosphorylation of PI3K and AKT was decreased markedly after MCAO operation. Treatment with miR-98 agomir and RiPerC operation significantly decreased PIK3IP1 and increased the expression of p-PI3K and p-AKT. However, RiPerC operation group treatment with miR-98 antagomir decreased the expression of p-PI3K and p-AKT. Therefore, PIK3IP1 induce apoptosis by inhibiting the PI3K/AKT signaling pathway.

In conclusion, our results indicate that RiPerC operation significantly decreased the infarct volume; improved sensorimotor function; reduced the IL-1*β*, IL-6, and TNF-*α* level and PIK3IP1 expression; and improved the expression of miR-98. Furthermore, injected with miR-98 antagomir can offset the cerebral ischemia-reperfusion injury relief by RiPerC treatment. Therefore, our finding indicates that RiPerC inhibited the expression of PIK3IP1 by upregulated miR-98, thereby reducing the apoptosis induced by PIK3IP1 through the PI3K/AKT signaling pathway, thus reducing the cerebral ischemia-reperfusion injury.

## Data Availability

The data used to support the finding of this study are available from the corresponding author upon request.
